# Metabolomics of Head and Neck Cancer: A Mini-Review

**DOI:** 10.3389/fphys.2016.00526

**Published:** 2016-11-08

**Authors:** Jae M. Shin, Pachiyappan Kamarajan, J. Christopher Fenno, Alexander H. Rickard, Yvonne L. Kapila

**Affiliations:** ^1^Department of Biologic and Materials Sciences, University of Michigan School of DentistryAnn Arbor, MI, USA; ^2^Department of Epidemiology, University of Michigan School of Public HealthAnn Arbor, MI, USA; ^3^Department of Periodontics and Oral Medicine, University of Michigan School of DentistryAnn Arbor, MI, USA; ^4^Division of Periodontology, Department of Orofacial Sciences, University of California San FranciscoSan Francisco, CA, USA

**Keywords:** head and neck cancer, oral cancer, squamous cell carcinoma, metabolomics, microbiome

## Abstract

Metabolomics is used in systems biology to enhance the understanding of complex disease processes, such as cancer. Head and neck cancer (HNC) is an epithelial malignancy that arises in the upper aerodigestive tract and affects more than half a million people worldwide each year. Recently, significant effort has focused on integrating multiple “omics” technologies for oncological research. In particular, research has been focused on identifying tumor-specific metabolite profiles using different sample types (biological fluids, cells and tissues) and a variety of metabolomic platforms and technologies. With our current understanding of molecular abnormalities of HNC, the addition of metabolomic studies will enhance our knowledge of the pathogenesis of this disease and potentially aid in the development of novel strategies to prevent and treat HNC. In this review, we summarize the proposed hypotheses and conclusions from publications that reported findings on the metabolomics of HNC. In addition, we address the potential influence of host-microbe metabolomics in cancer. From a systems biology perspective, the integrative use of genomics, transcriptomics and proteomics will be extremely important for future translational metabolomic-based research discoveries.

## Introduction

The incidence of head and neck cancer (HNC) exceeds half a million cases annually worldwide and accounts for approximately 3% of adult malignancies (Johnson et al., 2011; National Cancer Institute, 2013). HNC is defined as epithelial malignancies that arise in the aerodigestive tract (paranasal sinuses, nasal and oral cavity, pharynx and larynx) and can metastasize to different locations (Rezende et al., 2010). About 75% of HNCs are oral cancers and 90% of oral cancers are diagnosed as oral squamous cell carcinomas (OSCC) (Rezende et al., 2010; National Cancer Institute, 2013). Despite therapeutic and technological advances, the prognosis for HNC has not improved in decades due to its malignant and recurrent properties (Forastiere et al., 2001; Mao et al., 2004). The most widely accepted risk factors for HNC include tobacco (smoked or chewed), alcohol use, and human papillomavirus (HPV) infection (Gillison, 2004; Schmidt et al., 2004). However, these risk factors alone cannot explain the observed incidence and pathogenesis of HNC, since some patients are not in these risk categories. Thus, it is likely that other unknown factors play important roles in tumorigenesis, tumor progression and metastasis of HNC.

There has been an increasing trend to incorporate “omics” technology, including metabolomics, into oncological research (Vucic et al., 2012; Cho, 2013; Armitage and Barbas, 2014; Yu and Snyder, 2016). Investigators have explored different technologies and analytical methods to better understand the metabolomic properties of cancers, including HNC (Bathen et al., 2010; Blekherman et al., 2011; Beger, 2013; Liesenfeld et al., 2013; Olivares et al., 2015). As more independent reports on metabolomics of HNC are being published, a comprehensive meta-analysis of these large “omics” data sets will be of potential value in the near future to enhance translational studies. Specifically, metabolomic studies can help to potentially identify clinically relevant biomarkers that may be useful in early detection of cancer, to enhance the accuracy of diagnosis and prognosis, and to aid in the development of new drug targets to help improve therapeutic outcomes (Olivares et al., 2015; Yu and Snyder, 2016).

The objective of this mini-review is to summarize and discuss the published studies on HNC metabolomics. We will discuss the different technological tools utilized in metabolomics, and focus on the findings from studies that used different types of patient samples (i.e., saliva, serum, blood, urine, tissues). In addition to the host-metabolomic profiles, we discuss the potential relationship and influence of the microbial metabolome in cancers. By coupling metabolomics data with other omics data, we can achieve a greater understanding of complex cancer processes and derive new information that may help to better target aggressive and malignant cancer types, such as HNC.

### Biological samples used for head and neck cancer metabolomics

A broad array of biological fluids, such as saliva, blood and urine have been used in metabolomic-based studies (Nagana Gowda et al., 2008; Psychogios et al., 2011; Bouatra et al., 2013; Dame et al., 2015). These biofluids contain hundreds to thousands of detectable metabolites that can be obtained non- or minimally invasively (Beger, 2013). In addition, cell and tissue extracts can be a source of samples for metabolomic-based studies (Beger, 2013). With current diagnostic procedures requiring a tissue biopsy, a portion of the tissue samples can be harvested for further metabolomic analyses. The following discussion will focus on the findings, postulated hypotheses, and conclusions from the published metabolomic studies that used different biofluids and cell/tissue extracts to study HNC metabolomics.

### Saliva metabolomics

Saliva is an important biological fluid required for multiple functions, including speech, taste, digestion of foods, antiviral and antibacterial protection, to maintain adequate oral health (Loo et al., 2010; Spielmann and Wong, 2011). Saliva is readily available, and the collection process is simple and non-invasive. Thus, saliva has been a popular medium for “omics” based research studies (Zhang et al., 2012; Cuevas-Córdoba and Santiago-García, 2014). Two types of saliva that can be used for metabolomics studies are stimulated and unstimulated whole saliva. These two saliva types vary in their chemical composition, so it is important to identify the specific type of saliva that was used for the study (Humphrey and Williamson, 2001; Carpenter, 2013; Cuevas-Córdoba and Santiago-García, 2014).

Amongst different HNC types, OSCC is associated with a high morbidity rate and a poor 5-year survival rate of less than 50% (Epstein et al., 2002; Mao et al., 2004). To improve the prognosis for HNC, investigators have proposed using saliva metabolites to differentiate between precancerous and malignant lesions. Using hierarchical principal component analysis (PCA) and discriminate analysis algorithms, Yan and colleagues were able to distinguish between OSCC and its precancerous lesions oral lichen planus (OLP) and oral leukoplakia (OLK) (Yan et al., 2008; Table 1). Although the OLP and OLK groups were not as well separated in the PCA plot, the OSCC group showed a clear separation from the healthy and precancerous groups (Yan et al., 2008). In addition, Wei and others used ultra-performance liquid chromatography coupled with quadrupole/time-of-flight spectrometry (UPLC-QTOFMS) analysis to identify a signature panel of salivary metabolites that could distinguish OSCC from healthy controls (Wei et al., 2011; Table 1). Wei selected a panel of five salivary metabolites, which included γ-aminobutyric acid, phenylalanine, valine, n-eicosanoic acid and lactic acid. This combination of metabolites accurately predicted and distinguished OSCC from the control samples, suggesting that metabolomic approaches could complement the clinical detection of OSCC for improved diagnosis and prognosis (Wei et al., 2011).

**Table 1:** **Summary of metabolomic-based studies on head and neck cancers**.

**Subjects**	**Cancer**	**Sample**	**Detection method**	**Metabolomic findings**	**References**
50 HNSCC 77 healthy	HNSCC	Saliva	HPLC	Increased: Glutathione	Almadori et al., 2007
20 OSCC, 20 OLP 7 OLK 11 healthy	OSCC OLP OLK	Saliva	HPLC/MS	Metabolic profiling data distinguished between OSCC, OLP and OLK	Yan et al., 2008
69 oral cancer patients 87 healthy	Oral cancer	Saliva	CE-TOF-MS	28 differentially expressed metabolites were detected and was used to predict oral cancer outcome	Sugimoto et al., 2010
37 OSCC 32 oral leukoplakia 34 healthy	OSCC Oral leukoplakia	Saliva	UPLC-QTOFMS	41 metabolites distinguished OSCC from control, 61 distinguished OSCC from OLK, and 27 distinguished OLK from control	Wei et al., 2011
33 OSCC 5 OLK 28 healthy	OSCC OLK Healthy	Blood (plasma)	^1^H NMR	At least 17 metabolites were differentially expressed and differentiated OSCC from healthy	Zhou et al., 2009
15 OSCC 10 healthy	OSCC	Blood (serum)	1D ^1^H and 2D ^1^H J-resolved NMR	Altered energy metabolism: Lipolysis (increased levels of ketone bodies) TCA cycle (i.e., ↓citrate, succinate, formate) Amino acid catabolism (i.e., ↑ 2-hydroxbutyrate, ornithine, asparagine)	Tiziani et al., 2009
25 HNSCC (Of these patients, 17 used for serum and 19 used for tissue analysis)	HNSCC	Blood (serum) Tissues	GC/MS	Serum: ↑ Glycolysis, ↓ Amino acids Tissues ↑ Amino acids, ↓ Glycolysis	Yonezawa et al., 2013
37 OSCC 32 OLK 34 healthy	OSCC OLK	Urine	GC-MS	Increased: Alanine, tyrosine, valine, serine, and cysteine Decreased: Hippurate and 6-hydroxynicotic acid Regression model based on valine and 6-hydroxynicotic acid yielded an accuracy of 98.9%, sensitivity of 94.4%, specificity of 91.4%, and positive predictive value of 91.9% in distinguishing OSCC from the controls	Xie et al., 2012
*In vitro*: 19 HNSCC 13 healthy 3 metastatic cervical lymph node SCC cell line *In vivo*: 7 HNSCC 7 healthy	HNSCC	Tissues	^1^H MRS	Mean choline/creatine ratio was higher in HNSCC samples. Several amino acids including alanine, isoleucine, glutathione, histidine, valine, lysine and polyamine were differentially found in HNSCC samples	Mukherji et al., 1997
85 HNSCC 50 healthy	HNSCC	Tissues	^1^H MRS	Increased: Taurine, choline, glutamic acid, lactic acid, lipid	El-Sayed et al., 2002
159 OSCC (Tumor and neighboring margins and bed tissues)	OSCC	Tissues	HR-MAS NMR	Increased: Acetate, glutamate, lactate, choline, phosphocholine, glycine, taurine, leucine, isoleucine, valine, lysine, and alanine Decreased: Creatine, polyunsaturated fatty acids	Srivastava et al., 2011
22 HNSCC (matched samples divided into 18NAT, 18 tumor and 7 LN-Met)	HNSCC	Tissues	HR-MAS ^1^H NMR	HNSCC and LN-Met tissues showed elevated levels of lactate, amino acids and decreased levels of triglycerides	Somashekar et al., 2011
5 HNSCC cell lines 3 primary normal human oral keratinocytes from patients	HNSCC	Cells	^1^H NMR	21 differentially expressed metabolites: Increased: Lactate, isoleucine, valine, alanine, glutamine, glutamate, aspartate, glycine, phenylalanine, tyrosine, choline-containing compounds, creatine, taurine, glutathione Decreased: Triglycerides	Tripathi et al., 2012
2 cell lines (HNSCC cells and stem-like cancer cells)	HNSCC	Cells	Cap IC-MS	Changes in energy metabolism pathways: Glycolysis and TCA cycle	Wang et al., 2014

Cap IC-MS, Capillary anion exchange ion chromatography-mass spectrometry; CE-TOF/MS, Capillary electrophoresis-time-of-flight mass spectrometry; GC/MS, Gas chromatography/mass spectrometry; ^1^H-NMR, Proton nuclear magnetic resonance; HR-MAS; High resolution magic angle spinning; ^1^H-MRS, Proton magnetic resonance spectroscopy; HPLC, High performance liquid chromatography; LC/GC, Liquid chromatography/gas chromatography; NMR, Nuclear magnetic resonance; UPLC-QTOFMS, Ultra-performance liquid chromatography coupled with quadrupole/time-of-flight spectrometry; LN-Met, lymph node metastasis.

Work presented by Almadori and colleagues discovered that salivary glutathione (antioxidant), but not uric acid (antioxidant), was significantly increased in patients with oral and pharyngeal SCC compared to healthy controls (Almadori et al., 2007; Table 1). However, although there were significant alterations in the glutathione levels potentially due to metabolism of malignant cells, the concentrations were too inconsistent to suggest glutathione as a definitive SCC diagnostic marker (Almadori et al., 2007). Furthermore, Sugimoto and colleagues identified 28 metabolites that correctly differentiated oral cancers from control samples in their study (Sugimoto et al., 2010). Among these differentially expressed metabolites, salivary polyamine levels were markedly higher in oral cancer samples compared to other cancer samples (breast and pancreatic) and controls (Sugimoto et al., 2010). Polyamines are small molecules derived from amino acids that are essential for many biological functions (Dimery et al., 1987; Pegg, 2009). Increased polyamine levels have been associated with increased cell proliferation, decreased apoptosis and elevated expression of genes affecting tumor invasion and metastasis (Gerner and Meyskens, 2004). Thus, it is hypothesized that polyamine homeostasis is important for regulation of cancer related functions, such as cell proliferation and apoptosis.

Based on published studies that analyzed the salivary metabolome of HNC, there is a general consensus that unique metabolites specific to HNC exist. However, due to differences in detection and analytical methods, the current data still lacks coherency, and a common HNC metabolomic signature has yet to be identified.

### Blood and urine metabolomics

In addition to saliva, blood and urine are commonly used for metabolomic-based studies (Psychogios et al., 2011; Bouatra et al., 2013). Blood is divided into plasma—a cellular portion containing red and white blood cells and platelets, and serum—a non-cellular protein-rich liquid separately obtained following blood coagulation. Both plasma and serum contain a wide variety of metabolites, and current studies suggest that plasma and serum are similar in terms of metabolite content within the aqueous phase (Psychogios et al., 2011). Importantly, numerous studies have demonstrated that an altered chemical and protein metabolic composition can now be detected in blood samples obtained from subjects with pathology or diseases, such as cancer (Psychogios et al., 2011; DeBerardinis and Thompson, 2012). Tiziani and colleagues reported that OSCC patients exhibited abnormal metabolic activity in blood serum, wherein altered activity related to lipolysis, the TCA cycle and amino acid catabolism was detected (Tiziani et al., 2009; Table 1). For example, there was an increased level of ketone bodies present in OSCC samples, suggesting that increased lipolysis was a backup mechanism for energy production (Tiziani et al., 2009). Furthermore, a common signature for many cancers includes a high rate of glycolysis followed by lactic acid fermentation in the cytosol, rather than by a comparatively low rate of glycolysis followed by oxidation of pyruvate in the mitochondria, known as the “Warburg effect.” Similarly in HNC, Tiziani demonstrated that OSCC tumors relied heavily on glycolysis as a main energy source (Warburg, 1956; Tiziani et al., 2009).

**Figure 1:**
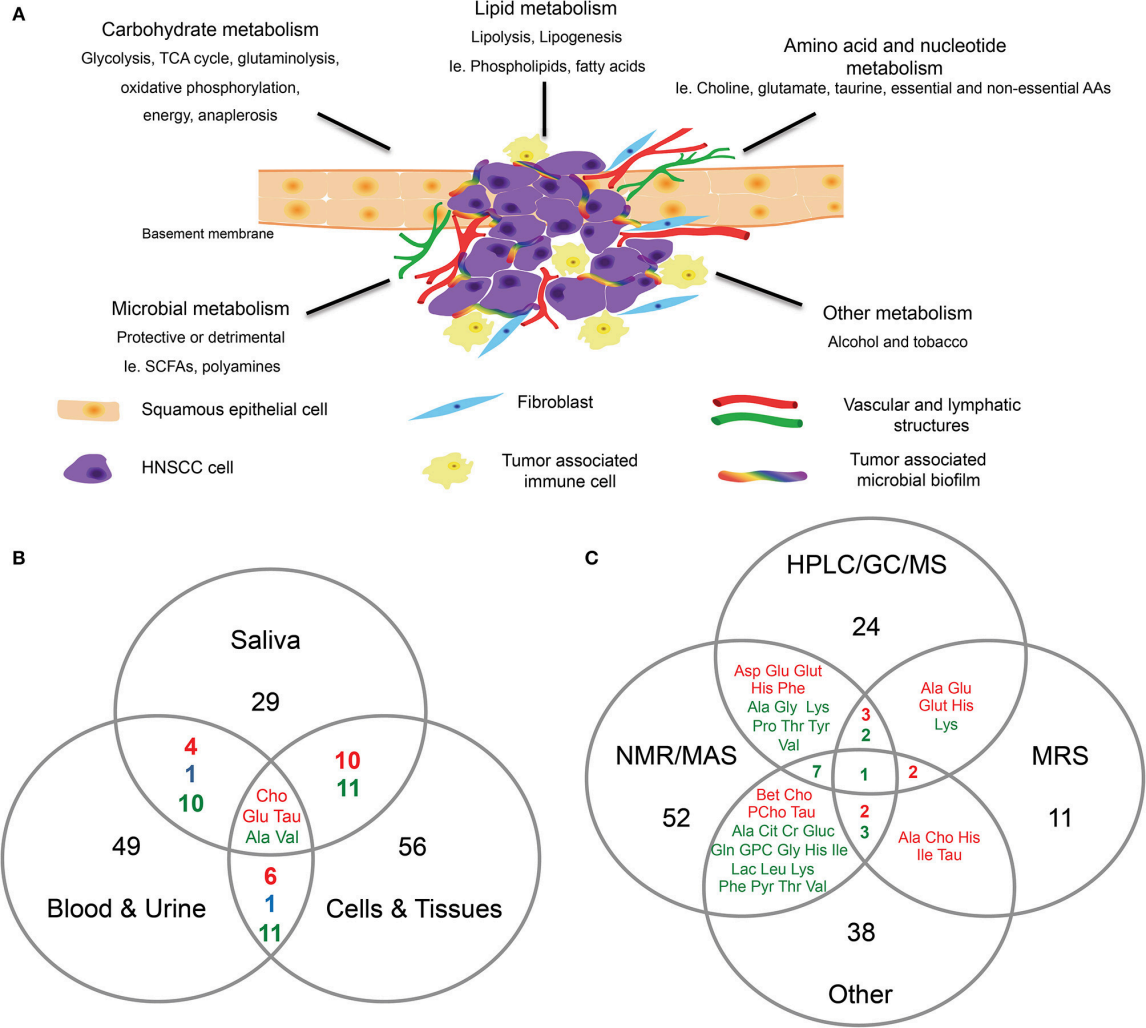
**Head and neck cancer metabolism. (A)** Proposed schematic representation of HNC tumor microenvironment. Altered metabolism in HNC can result in differential expression of metabolites associated with carbohydrates, lipids, amino acids, and nucleotide metabolism. The co-inhabiting microbiota of the TME can further result in altered metabolic activity. In addition to the genomic transformation of cancer cells, diet and lifestyle (alcohol, tobacco) are risk factors contributing to the altered cancer metabolism. **(B,C)** Venn diagrams showing, **(B)** Overlap of differentially expressed metabolites identified in HNC in saliva, blood and urine, and cells and tissues. **(C)** Overlap of differentially expressed metabolites in HNC identified by different detection methods such as HPLC/GC/MS, NMR/MAS, MRS and other. Metabolites were selected and compiled from studies in Table 1. Red, detected in increased levels; Blue, detected in decreased levels; Green, detected in increased and decreased levels.

Yonezawa and others identified several metabolites that were altered in serum and tissue samples of HNSCC patients who experienced relapse (Yonezawa et al., 2013). The four metabolites that were significantly altered were glucose, methionine, ribulose, and ketoisoleucine (Yonezawa et al., 2013). Interestingly, when the authors compared the metabolomic profiles of the OSCC serum and tissue samples, an inverse relationship was observed in the differentially expressed metabolites (Yonezawa et al., 2013; Table 1). Metabolites associated with glycolytic pathways (i.e., glucose) were lower in the tissues, whereas amino acids (i.e., valine, tyrosine, serine, and methionine) were expressed in higher levels in the tissues than the serum (Yonezawa et al., 2013). In addition, the serum metabolomic profiles differed between patients with or without HNSCC relapse (Yonezawa et al., 2013). Several other studies further support that serum and plasma samples from HNC subjects possess distinct metabolomic profiles. For example, elevated levels of choline-containing compounds were detected in OSCC samples in numerous studies (Maheshwari et al., 2000; El-Sayed et al., 2002; Bezabeh et al., 2005; Tiziani et al., 2009; Zhou et al., 2009). Choline is an important constituent of phospholipid metabolism in cellular membranes and is considered a biomarker for cancer cell proliferation, survival and malignancy (Ackerstaff et al., 2003; Glunde et al., 2006, 2011). Through our comprehensive analysis, choline was identified as one of the metabolites that was consistently over expressed in HNC samples regardless of sample types (Figure 1B). Studies have suggested a link between cancer feedback cell signaling and choline metabolism (Aboagye and Bhujwalla, 1999; Ackerstaff et al., 2003; Janardhan et al., 2006; Glunde et al., 2011; Ridgway, 2013). Thus, an abnormal choline metabolism in cancer has gained much attention and is regarded as a metabolic hallmark for tumor development and progression (Glunde et al., 2011).

The use of urine samples in HNC metabolomic studies is not as common compared to the other types of biofluids mentioned above. However, urine is widely used by metabolomic researchers for other conditions or diseases due to its ease of collection and the wide coverage of metabolites that is possible with urine samples (Bouatra et al., 2013). Thus far, there has only been a single study reported on HNC metabolomics using urine. From patient urine samples, Xie and colleagues identified a panel of differentially expressed metabolites and demonstrated their utility by logistic regression (LR) modeling (Xie et al., 2012; Table 1). When two metabolites, valine and 6-hydroxynicotic acid, were inputted together in the LR prediction model,the authors were able to identify OSCC with a 98.9% accuracy, and a greater than 90% sensitivity, specificity and positive predictive value (Xie et al., 2012). However, similar to saliva and blood metabolomics, the use of urine samples for HNC metabolomics will require further validation through more independent studies.

### Cell and tissue metabolomics

The current gold standard for diagnosis of HNC is a scalpel-obtained biopsy and subsequent histopathological interpretation. However, the current procedure is subjective and does not capture the full heterogeneic properties of neoplastic processes, as it is difficult to distinguish between precancerous from cancerous and malignant lesions (Rezende et al., 2010; Yu and Snyder, 2016). Early studies with magnetic resonance spectroscopy (MRS) using patient tissue samples demonstrated that a higher choline to creatine ratio was observed in HNC samples compared to healthy controls (Mukherji et al., 1997; El-Sayed et al., 2002; Table 1). In addition, Mukherji and colleagues reported that elevated levels of amino acids, such as alanine, glutathione, histidine, isoleucine, valine, lysine, and polyamines were more likely found in tumors compared to controls, and similar metabolites, such as glutathione and polyamines were also elevated in saliva associated with HNC (Mukherji et al., 1997; Almadori et al., 2007; Sugimoto et al., 2010). Srivastava and others used proton high-resolution magic angle spinning magnetic resonance (HR-MAS MR) spectroscopy to identify the metabolic perturbations of OSCC tumors compared to healthy controls. The data revealed higher levels of lactate, phosphocholine, choline and amino acids, and decreased levels of PUFA and creatine in OSCC samples compared to non-malignant samples (Srivastava et al., 2011). As previously mentioned, higher levels of detected choline in HNC tissues may indicate increased cancer cell proliferation and membrane biosynthesis, as a result of reciprocal interactions between oncogenic signaling and choline metabolism (Glunde et al., 2011). The reduced level of creatine could also be an indication of increased energy metabolism in tumors (Mukherji et al., 1997; El-Sayed et al., 2002).

Somashekar and colleagues reported that tumorous tissues biopsied from different anatomical locations (tongue, lip, oral cavity, and larynx) displayed similar metabolomic profiles between one another, suggesting that HNSCC tissues share similar metabolic activity during malignant transformation (Somashekar et al., 2011; Table 1). Primary and metastatic HNSCC tissues both showed increased/altered levels of branched chain amino acids, lactate, alanine, glutamine, glutamate, glutathione, aspartate, creatine, taurine, phenylalanine, tyrosine and choline compounds, with decreased levels of triglycerides (Somashekar et al., 2011; Table 1). In addition, Tripathi and others demonstrated that the cell extracts of HNSCC displayed comparable metabolic phenotypes as observed in the HNSCC tissues (Tripathi et al., 2012; Table 1). Thus, based on published reports, the metabolites associated with malignant transformation of HNC are associated with multiple dysregulated metabolic pathways, including glycolysis, glutaminolysis, oxidative phosphorylation, energy metabolism, TCA cycle, osmo-regulatory and anti-oxidant mechanisms (Figure 1; Somashekar et al., 2011; Tripathi et al., 2012; Wang et al., 2014).

### Influence of microbial metabolomics

The human body is a host to taxonomically diverse multi-species microbial communities. In particular, the oral cavity and the gut are home to hundreds of transient and resident microbial species (Eckburg et al., 2005; Dewhirst et al., 2010). Several publications suggest that the microbiota that colonize the human body (particularly the oral cavity and gut) contribute to the etiology of different types of cancers because of their ability to alter the community composition and induce inflammatory reactions, DNA damage and apoptosis, and an altered metabolism (Meurman, 2010; Chen et al., 2012; Farrell et al., 2012; Louis et al., 2014). Thus, when considering cancer-associated metabolomics, the influence of the microbiota and its repertoire of metabolites should also be considered, since the microbiota are profoundly abundant in the human body and cancerous tissues.

Colorectal cancer (CRC), like HNC, is associated with risk factors that include diet and lifestyle (Gingras and Béliveau, 2011). Specific bacterial genera, like *Fusobacterium*, are found in greater abundance in patients diagnosed with CRC, colorectal adenomas, pancreatic cancer and HNC (Castellarin et al., 2012; Farrell et al., 2012; Kostic et al., 2012; McCoy et al., 2013). Accumulated data suggest that diverse polymicrobial communities can produce a wide range of metabolites by metabolic fermentation (Tang, 2011). For instance, gut microorganisms can secrete a variety of metabolites that may play a role in the etiology and prevention of complex diseases (Heinken and Thiele, 2015). These microbial metabolites can directly regulate and modulate the host-tumor cell metabolism (Figure 1A); bacteria isolated from the gut can produce metabolites that are protective or detrimental to the host tissues and cells. For example, short-chain fatty acids (SCFAs) like butyrate, acetate, and propionate function in the suppression of inflammation and cancer, whereas other metabolites, such as polyamines, are toxic and cancer-promoting at high levels (Louis et al., 2014). Alterations in microbial diversity and function due to known risk factors for HNC (alcohol and tobacco use) and unknown factors could actively contribute to HNC tumorigenesis (Schwabe and Jobin, 2013; Figure 1A).

## Concluding remarks

The complement of “omics” based approaches could significantly enhance our understanding of the complex processes of HNC tumorigenesis. Although, it is extremely complex, progress has been made in integrating two or more omics data sets to study cancer (Cho, 2013). For example, studies have examined the molecular differences between HPV+ and HPV− HNCs by comparing the differences in their genomic, transcriptomic, and proteomic profiles (Sepiashvili et al., 2015). Since Otto Warburg's first hypothesis of the altered metabolism of cancer cells, the field of cancer metabolomics has rapidly expanded and revealed intriguing new data regarding metabolic pathways associated with cancers (Warburg, 1956). With fast-moving advancements in technology and bioinformatics, the quality of data output and the ability to detect small molecular metabolites has significantly improved. Thus, investigators will likely soon be able to transition from untargeted global metabolomic approaches to more focused targeted and mechanistic-based metabolomic studies. In addition, with the availability of growing public databanks, investigators can now search for specific omics variations that characterize different types of cancers and phenotypes of a cancer (Cho, 2013).

From the clinical perspective, understanding the metabolic pathways associated with life threatening conditions, such as cancer, could be extremely valuable in decreasing the burden of disease. With saliva-based DNA screening tests already available for chair-side use in dentistry for HNC, we can envision a saliva-based screening or diagnostic test that incorporates omics that replaces the surgical biopsy and provides a more individualized and robust patient health, disease, or risk profile. Here, we discussed the metabolomics of both the host (normal and cancerous conditions) and co-existing microbiota (Figure 1A). In addition, we organized the differentially expressed metabolites from previous publications by sample types (saliva, blood and urine, cells and tissues) and detection methods (Figures 1B,C). The full integration and routine inclusion of metabolomics in the clinic has yet to be implemented, however, continued research and translational efforts will reinforce the promise of this evolving technology and science. Studies to date have been conducted with relatively small patient sample sizes, with different sample types and detection methods. In the future, it will be critical to follow up with larger, more comprehensive population studies to confirm the validity of the current findings. In addition, sharing detailed sample collection and analytical methods between investigators will be essential to conduct sound HNC metabolomics research. From the systems biology perspective, the integration of other omics data with metabolomics data will be required for a greater understanding of cancer biology.

## Author contributions

JS wrote the manuscript, put the figure and table together, and edited the manuscript. PK, JF, and AR edited the manuscript, edited the figure and table, and edited the manuscript. JF, AR, and YK conceived the topic for the mini review, assisted with the manuscript writing, assisted with the figure and table and edited the manuscript.

### Conflict of interest statement

The authors declare that the research was conducted in the absence of any commercial or financial relationships that could be construed as a potential conflict of interest.

## Abbreviations

Ala: (alanine)
Asp: (aspartate)
Bet: (betaine)
Cit: (citrate)
Cr: (creatinine)
Cho: (choline)
Glu: (glutamate)
Gluc: (glucose)
Gln: (glutamine)
Glut: (glutathione)
Gly: (glycine)
GPC: (glycerophosphocholine)
His: (histidine)
Ile: (isoleucine)
Lac: (lactate)
Leu: (leucine)
Lys: (lysine)
PCho: (phosphocholine)
Phe: (phenylalanine)
Pro: (proline)
Pyr: (pyruvate)
Tau: (taurine)
Thr: (threonine)
Tyr: (tyrosine)
Val: (valine).
